# Fenofibrate Protects against Retinal Dysfunction in a Murine Model of Common Carotid Artery Occlusion-Induced Ocular Ischemia

**DOI:** 10.3390/ph14030223

**Published:** 2021-03-07

**Authors:** Deokho Lee, Yohei Tomita, Yukihiro Miwa, Heonuk Jeong, Kiwako Mori, Kazuo Tsubota, Toshihide Kurihara

**Affiliations:** 1 Laboratory of Photobiology, Keio University School of Medicine, Tokyo 160-8582, Japan; deokholee@keio.jp (D.L.); yohei.tomita@childrens.harvard.edu (Y.T.); yukihiro226@gmail.com (Y.M.); jeong.h@keio.jp (H.J.); morikiwako@gmail.com (K.M.); 2 Department of Ophthalmology, Keio University School of Medicine, Tokyo 160-8582, Japan; 3 Boston Children’s Hospital/Harvard Medical School, Boston, MA 02115, USA; 4 Animal Eye Care, Tokyo Animal Eye Clinic, Tokyo 158-0093, Japan; 5 Tsubota Laboratory, Inc., Tokyo 160-0016, Japan

**Keywords:** common carotid artery, electroretinography, fibroblast growth factor 21, fenofibrate, peroxisome proliferator-activated receptor alpha

## Abstract

Ocular ischemia is a common cause of blindness and plays a detrimental role in various diseases such as diabetic retinopathy, occlusion of central retinal arteries, and ocular ischemic syndrome. Abnormalities of neuronal activities in the eye occur under ocular ischemic conditions. Therefore, protecting their activities may prevent vision loss. Previously, peroxisome proliferator-activated receptor alpha (PPARα) agonists were suggested as promising drugs in ocular ischemia. However, the potential therapeutic roles of PPARα agonists in ocular ischemia are still unknown. Thus, we attempted to unravel systemic and ocular changes by treatment of fenofibrate, a well-known PPARα agonist, in a new murine model of ocular ischemia. Adult mice were orally administered fenofibrate (60 mg/kg) for 4 days once a day, followed by induction of ocular ischemia by unilateral common carotid artery occlusion (UCCAO). After UCCAO, fenofibrate was continuously supplied to mice once every 2 days during the experiment period. Electroretinography was performed to measure retinal functional changes. Furthermore, samples from the retina, liver, and blood were subjected to qPCR, Western blot, or ELISA analysis. We found that fenofibrate boosted liver function, increased serum levels of fibroblast growth factor 21 (FGF21), one of the neuroprotective molecules in the central nervous system, and protected against UCCAO-induced retinal dysfunction. Our current data suggest a promising fenofibrate therapy in ischemic retinopathies.

## 1. Introduction

Central retinal artery occlusion (CRAO), first described by von Graefe in 1859, suddenly induces irreversible vision loss [1]. This disease is categorized as an ophthalmic and medical emergency, and 80% of CRAO patients have low visual acuity below 20/400 afterward [2]. The incidence of CRAO is around 1.9 in 100,000 and its incidence increases with aging [3,4]. The major causes of this disease are divided into two categories: non-arthritis and arteritis [5,6]. More than 90% of causes are non-arthritis such as carotid artery atherosclerosis, cardiogenic embolism, hematological conditions and other vascular diseases [7,8]. These factors could be induced by hypertension, diabetes mellitus, smoking, coronary artery disease, and cerebral vascular accidents [1]. It is important to control these diseases/injuries, as more people are affected by CRAO in an aging society. Although there are multiple choices for a rescue, results of current treatments are not always satisfactory. In fact, there is no standardized treatment for CRAO. Besides, precise mechanisms of this disease have not been fully understood yet.

Murine experimental models of carotid artery occlusion have been used to unravel pathological mechanisms for ocular ischemia [9,10,11,12,13,14]. Anatomically, the central retinal artery is derived from the ophthalmic artery, one of the branches of the internal carotid artery of the common carotid artery. Therefore, carotid artery occlusion could potentially induce CRAO, finally leading to ocular ischemia and vision loss [15,16]. Bilateral common carotid artery occlusion to induce ocular ischemia has been applied to rats, in that the circle of Willis in rats is well-organized [9,10,11,12,13,17]. However, several studies reported that bilateral common carotid artery occlusion could not be applicable to mice with a high rate of death (almost 100%) because of a lack of posterior communicating arteries in the circle of Willis in mice [14,18,19]. Hence, unilateral common carotid artery occlusion (UCCAO) has been applied to mice for understanding pathological mechanisms for ocular ischemia [14,20]. Although several phenotypes for ocular ischemia were described in this model [14], a rescue for ocular ischemia has not been extensively studied. In this regard, the development of a cure for ocular ischemia in this model could be of interest.

Large-scale clinical studies such as the Fenofibrate Intervention and Event Lowering in Diabetes (FIELD) study and the Action to Control Cardiovascular Risk in Diabetes (ACCORD) study have demonstrated that fenofibrate, a peroxisome proliferator-activated receptor alpha (PPARα) agonist, inhibited progression of diabetic ischemic retinopathy [21,22,23]. The FIELD study demonstrated that fenofibrate treatment reduced the amount of laser photocoagulation for proliferative diabetic retinopathy and diabetic macular edema [21]. The ACCORD study showed positive modulation of blood glucose levels and reduction of the rate of progression for diabetic retinopathy in subjects receiving fenofibrate and simvastatin, in comparison with those receiving placebo and simvastatin [24,25]. Multiple studies have suggested that PPARα agonists could become possible promising drugs for retinal ischemia [26,27], and activation of PPARα by fenofibrate has been shown to suppress pathological angiogenesis in the retina in vivo and in vitro [28]. However, protective effects of fenofibrate against UCCAO-induced ocular ischemic injuries have not been discovered.

In this study, we aimed to investigate protective roles of fenofibrate in a new murine model of ocular ischemia induced by UCCAO which could be considered as one type of human CRAO.

## 2. Results

### 2.1. UCCAO Induces Ocular Ischemia in Adult Mice

**Figure 1:**
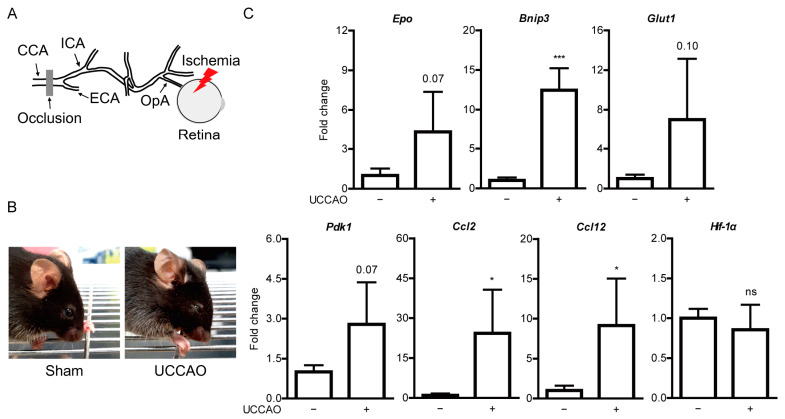
General features of ocular ischemia induced by unilateral common carotid artery occlusion (UCCAO) in adult mice. (**A**) Schematic illustration of induction of ocular ischemia by UCCAO. Ocular ischemia (a red thunder) can be induced by occlusion (a gray bar) of the common carotid artery (CCA) in that the ophthalmic artery (OpA) is originated from the internal carotid artery (ICA) of CCA. (**B**) Representative images show eyelid drooping in the eye 1 day after UCCAO. (**C**) Quantitative analyses (*n* = 4 per group) show increases in *Bnip3*, *Ccl2,* and *Ccl12* mRNA expressions in the UCCAO-operated retina 1 day after UCCAO, in comparison with those in the sham-operated retina. * *p* < 0.05, *** *p* < 0.001. The data were analyzed using Student’s t-test (two-tailed) and presented as mean ± standard deviation.

**Figure A1:**
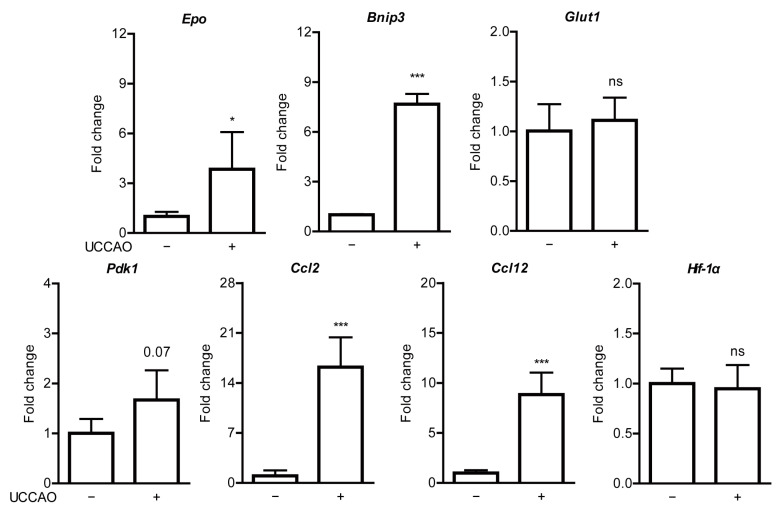
Increases in hypoxia-responsive gene expressions in the retina after UCCAO. Quantitative analyses (*n* = 4–7 per group) show increases in expressions of *Epo*, *Bnip3*, *Ccl2,* and *Ccl12* in the UCCAO-operated retina 2 days after UCCAO, in comparison with those in the sham-operated retina. * *p* < 0.05, *** *p* < 0.001. The data were analyzed using Student’s *t*-test (two-tailed) and presented as mean ± standard deviation. UCCAO; unilateral common carotid artery occlusion.

**Figure A2:**
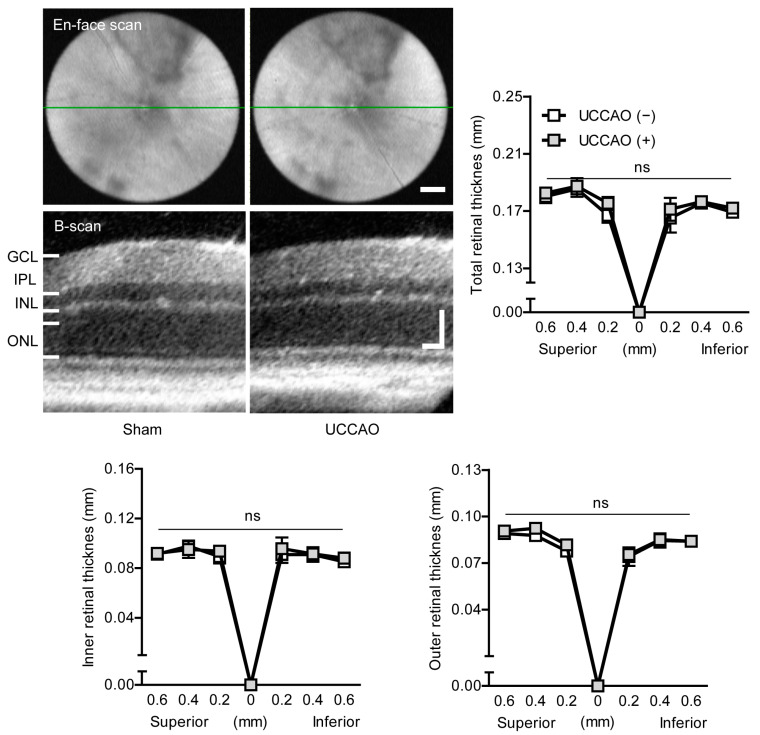
No change in retinal thickness after UCCAO. Representative optical coherence tomography (OCT) images (b-scan) in the sham- and UCCAO-operated retinas and quantitative analyses (*n* = 4–5 per group) show that there was no change in retinal thickness a week after UCCAO. The values in the horizontal axis of the graph represent 0.2, 0.4, and 0.6 mm distance from the optic nerve head (0) which was detected by the green line (en-face scan). Representative OCT images were taken at 0.4 mm from the optic nerve head. The data were analyzed using two-way ANOVA followed by a Bonferroni post hoc test and presented as a spider diagram (mean ± standard deviation). *p* > 0.05. GCL, IPL, INL, and ONL represent the ganglion cell layer, inner plexiform layer, inner nuclear layer, and outer nuclear layer, respectively. Scale bars are 200 (upper) and 50 (lower; vertical and horizontal bars) µm, respectively. UCCAO; unilateral common carotid artery occlusion.

**Figure A3:**
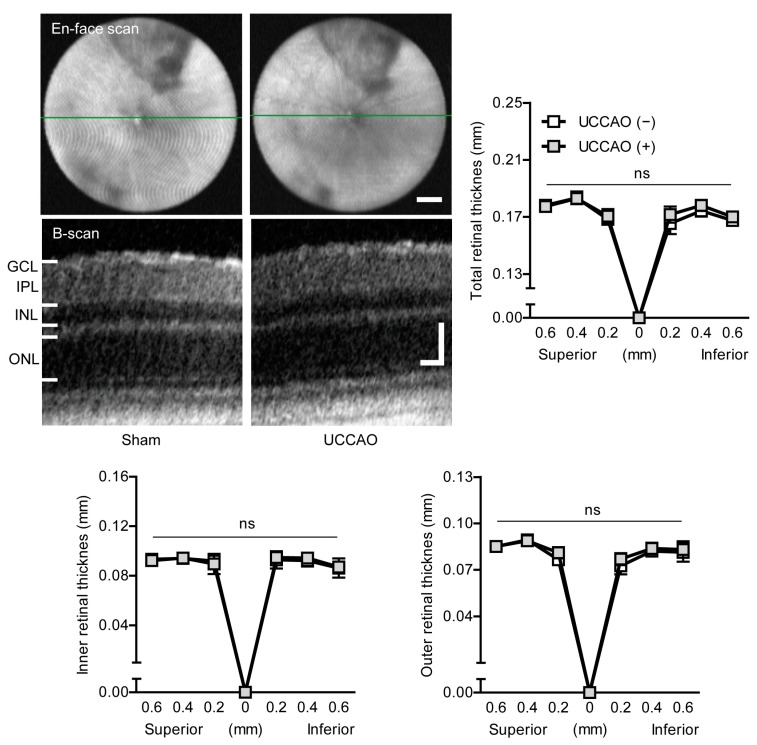
No change in retinal thickness after UCCAO. Representative optical coherence tomography (OCT) images (b-scan) in the sham- and UCCAO-operated retinas and quantitative analyses (*n* = 4–5 per group) show that there was no change in retinal thickness 7 weeks after UCCAO. The values in the horizontal axis of the graph represent 0.2, 0.4 and 0.6 mm distance from the optic nerve head (0) which was detected by the green line (en-face scan). Representative OCT images were taken at 0.4 mm from the optic nerve head. The data were analyzed using two-way ANOVA followed by a Bonferroni post hoc test and presented as a spider diagram (mean ± standard deviation). *p* > 0.05. GCL, IPL, INL, and ONL represent the ganglion cell layer, inner plexiform layer, inner nuclear layer, and outer nuclear layer, respectively. Scale bars are 200 (upper) and 50 (lower; vertical and horizontal bars) µm, respectively. UCCAO; unilateral common carotid artery occlusion.

To investigate protective roles of fenofibrate in the ischemic retina, we attempted to induce ocular ischemia in adult mice by ligation of the common carotid artery in the right side as mice could not survive after bilateral ligation of the common carotid arteries [14]. Ocular ischemia could be induced by occlusion of the common carotid artery as the ophthalmic artery is derived from the internal carotid artery of the common carotid artery (Figure 1A). We used the right eye of the UCCAO-operated mice for further analyses in this current study. As expected based on a previous study [14], we could observe eyelid drooping as a sign of successful occlusion (Figure 1B). Furthermore, we detected upregulation of hypoxia-responsive genes in the retina 1 day after the occlusion (Figure 1C). Significant increases were seen in *Bnip3*, *Ccl2,* and *Ccl12* (Figure 1C). Increasing tendencies were seen in expressions of *Epo*, *Glut1,* and *Pdk1* without statistical significance. There was no significant change in *Hif-1α* expression. 2 days after UCCAO, *Bnip3*, *Ccl2* and *Cc1l2* expressions were kept significantly upregulated and a significant increase in *Epo* expression was seen in the UCCAO-operated retina, in comparison with those in the sham-operated retina (Figure A1). *Pdk1*, *Glut1* and *Hif-1α* expressions were still not showing any significant change after UCCAO (Figure A1). Next, we investigated whether UCCAO induce retinal thinning in mice. We could not detect retinal thinning until 7 weeks after UCCAO, explained by no change in retinal thickness in the UCCAO-operated mice in comparison with that in the sham-operated mice (Figure A2 and Figure A3).

### 2.2. Fenofibrate Leads to PPARα Target Gene Expressions in Adult Mice

**Figure A4:**
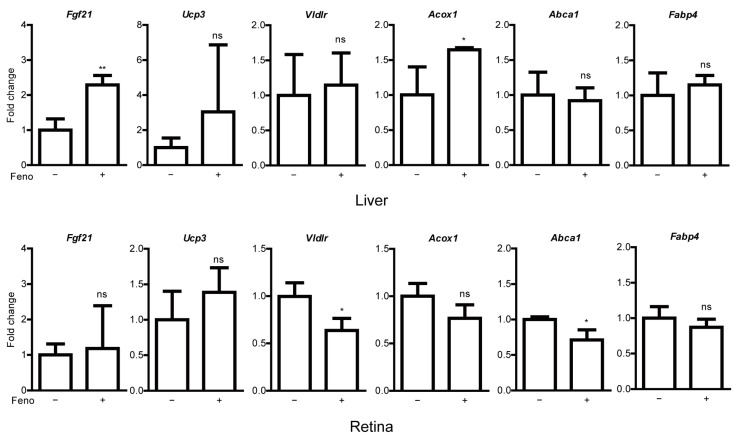
Increases in PPARα downstream gene expressions in the liver by oral administration of fenofibrate in adult mice. Quantitative analyses (upper; *n* = 3 per group) show that oral administration of fenofibrate for 4 days significantly increased *Fgf21* and *Acox1* expressions in the liver. Quantitative analyses (lower; *n* = 3 per group) show that oral administration of fenofibrate for 4 days showed significant decreases in expressions of *Vldlr* and *Abca1* in the retina. However, their decreasing fold changes were not dramatically seen. * *p* < 0.05, ** *p* < 0.01. The data were analyzed using Student’s *t*-test (two-tailed) and presented as mean ± standard deviation.

Duration of oral administration and concentration of fenofibrate was referred from previous papers demonstrating protective roles of fenofibrate against ischemia in the central nervous system with minor modification [29,30]. Oral administration of 60 mg/kg of fenofibrate once a day was applied to mice for 4 days using oral gavage needles. Then, we examined expressions of PPARα downstream genes in mice (Figure A4). The retina and the liver were used as expected target sites, in that the retina is our region of interest and the liver has been widely known to be associated with fenofibrate-related PPARα activation [31,32,33]. PPARα downstream genes such as *Fgf21* and *Acox1* were significantly upregulated in the fenofibrate-administered liver, compared with those in the vehicle-administered liver (Figure A4). Other genes such as *Ucp3*, *Vldlr*, *Abca1,* and *Fabp4* were not changed in the liver after 4 days of consecutive oral administration of fenofibrate. There was no significant change in *Fgf21*, *Ucp3*, *Acox1,* and *Fabp4* between the fenofibrate-administered retina and the vehicle-administered retina (Figure A4). Expressions of *Vldlr* and *Abca1* significantly decreased but their decreasing fold changes were not dramatic.

### 2.3. Fenofibrate Suppresses Retinal Dysfunction in a Mouse Model of UCCAO-Induced Ocular Ischemia

**Figure 2:**
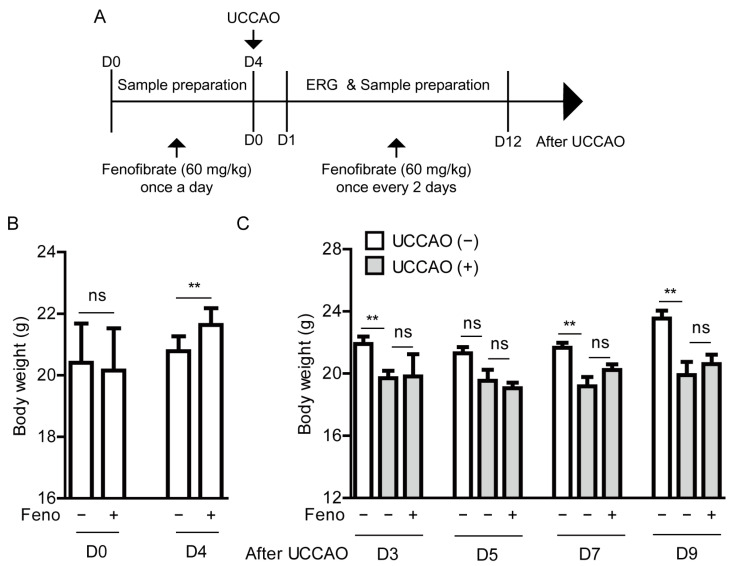
General observations for adult mice after oral administration of fenofibrate (**A**) A schematic illustration shows oral administration of fenofibrate to mice and a time point of induction of UCCAO and following experiments. ERG; electroretinography, UCCAO; unilateral common carotid artery occlusion. (**B**) Changes in body weight after oral administration of fenofibrate before UCCAO (*n* = 5–11 per group). (**C**) Changes in body weight after oral administration of fenofibrate after UCCAO (*n* = 5–6 per group). ** *p* < 0.01. The data were analyzed using Student’s *t*-test (two-tailed) or one-way ANOVA followed by a Bonferroni post hoc test, and presented as mean ± standard deviation. Feno; fenofibrate.

Based on our observation above, fenofibrate was initially supplied to mice via oral administration (60 mg/kg) once a day for 4 days before the occlusion (Figure 2A). Oral administration of fenofibrate slightly increased the body weight of adult mice (Figure 2B). After the occlusion, fenofibrate continued to be provided to mice via oral administration of fenofibrate once every 2 days (Figure 2A). Increased body weight was not seen in the fenofibrate-administered UCCAO-operated mice, in comparison with the vehicle-administered UCCAO-operated mice (Figure 2C). We only found that decreased body weight was seen in the UCCAO-operated mice, in comparison with the sham-operated mice.

**Figure 3:**
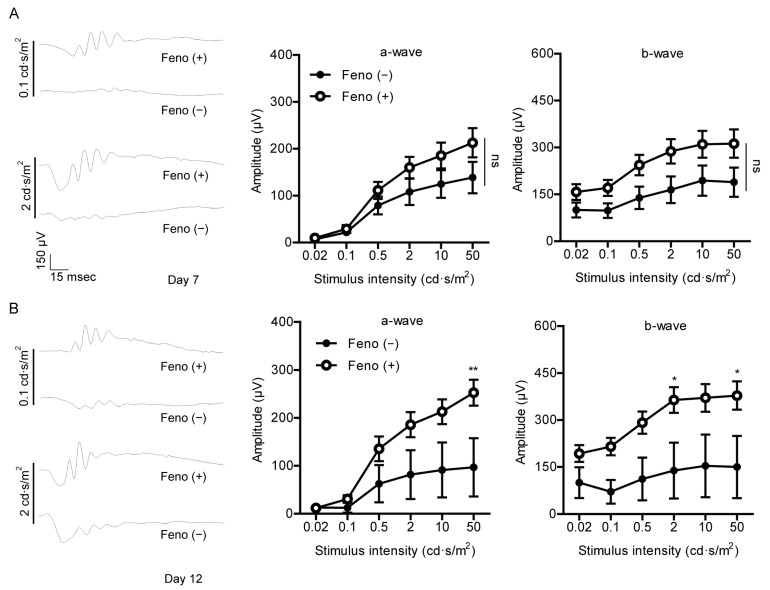
Protective effects of fenofibrate against retinal dysfunction induced by UCCAO. (**A**) Representative waveforms of a- and b-waves (low and high intensities, 0.1 and 2 cd·s/m^2^, respectively) and quantitative analyses (*n* = 9–11 per group) show that oral administration of fenofibrate slightly suppressed reduction in the amplitude of b-wave in the UCCAO-operated eye 7 days after UCCAO. (**B**) Representative waveforms of a- and b-waves (low and high intensities, 0.1 and 2 cd·s/m^2^, respectively) and quantitative analyses (*n* = 5–6 per group) show that oral administration of fenofibrate dramatically suppressed reduction in the amplitude of b-wave in the UCCAO-operated eye 12 days after UCCAO. * *p* < 0.05, ** *p* < 0.01. The data were analyzed using two-way ANOVA followed by a Bonferroni post hoc test and presented as mean ± standard error of the mean. Feno; fenofibrate. UCCAO; unilateral common carotid artery occlusion.

**Figure A5:**
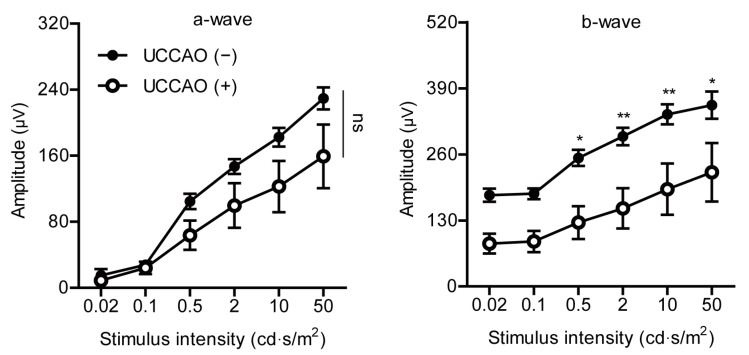
Retinal dysfunction induced by UCCAO. Quantitative analyses (*n* = 10 per group) show dramatic reduction in the amplitude of b-wave in the UCCAO-operated eye 7 days after UCCAO. * *p* < 0.05, ** *p* < 0.01. The data were analyzed using two-way ANOVA followed by a Bonferroni post hoc test and presented as mean ± standard error of the mean. UCCAO; unilateral common carotid artery occlusion.

**Figure A6:**
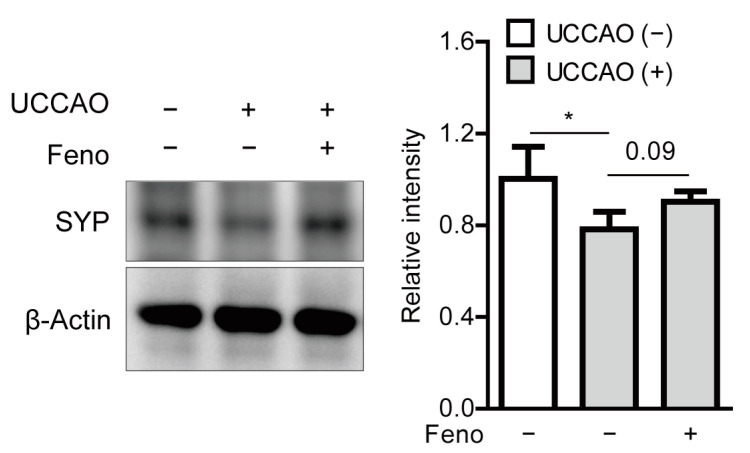
A slight increase in expression of retinal synaptophysin (SYP) by oral administration of fenofibrate in the UCCAO-operated mice. A representative image and quantitative analysis (n = 4–5 per group) show that SYP expression in the retina decreased 12 days after UCCAO and its reduction was slightly suppressed by oral administration of fenofibrate. * *p* < 0.05 (one-way ANOVA), *p* = 0.09 (Student’s *t*-test). The data were analyzed using one-way ANOVA followed by a Bonferroni post hoc test and Student’s *t*-test, and presented as mean ± standard deviation. Feno; fenofibrate. UCCAO; unilateral common carotid artery occlusion.

To determine protective effects of fenofibrate against retinal dysfunction in the UCCAO-operated mice, we performed ERG (Figure 3). Preliminarily, UCCAO induced retinal dysfunction, explained by a significant decrease in the amplitude of b-wave and a slight decrease in the amplitude of a-wave in the eye 7 days after UCCAO (Figure A5). Next, protective effects of fenofibrate were investigated under the same condition. We found that oral administration of fenofibrate slightly suppressed reduction in the amplitude of b-wave in the UCCAO-induced eye 7 days after UCCAO (Figure 3A). Furthermore, this protective effect was more dramatically shown 12 days after UCCAO (Figure 3B). Although there was no dramatic difference in the amplitude of a-wave between the fenofibrate-administered UCCAO-operated eye and the vehicle-administered UCCAO-operated eye, we could detect an increase in the amplitude of a-wave in the fenofibrate-administered UCCAO-operated eye both 7 and 12 days after UCCAO (Figure 3). Next, we examined whether its protection could be associated with regulation of the synaptic vesicle protein, synaptophysin, as its expression has been reported to be abundant in the inner retinal layer [34]. Decreased synaptophysin expression was seen in the retina 12 days after UCCAO (Figure A6). Although there was no statistical significance, we could see a reduction in synaptophysin expression was slightly suppressed in the fenofibrate-administered UCCAO-operated retina (Figure A6).

### 2.4. Fenofibrate Induces PPARα Target Gene Expressions in a Mouse Model of UCCAO-Induced Ocular Ischemia

**Figure 4:**
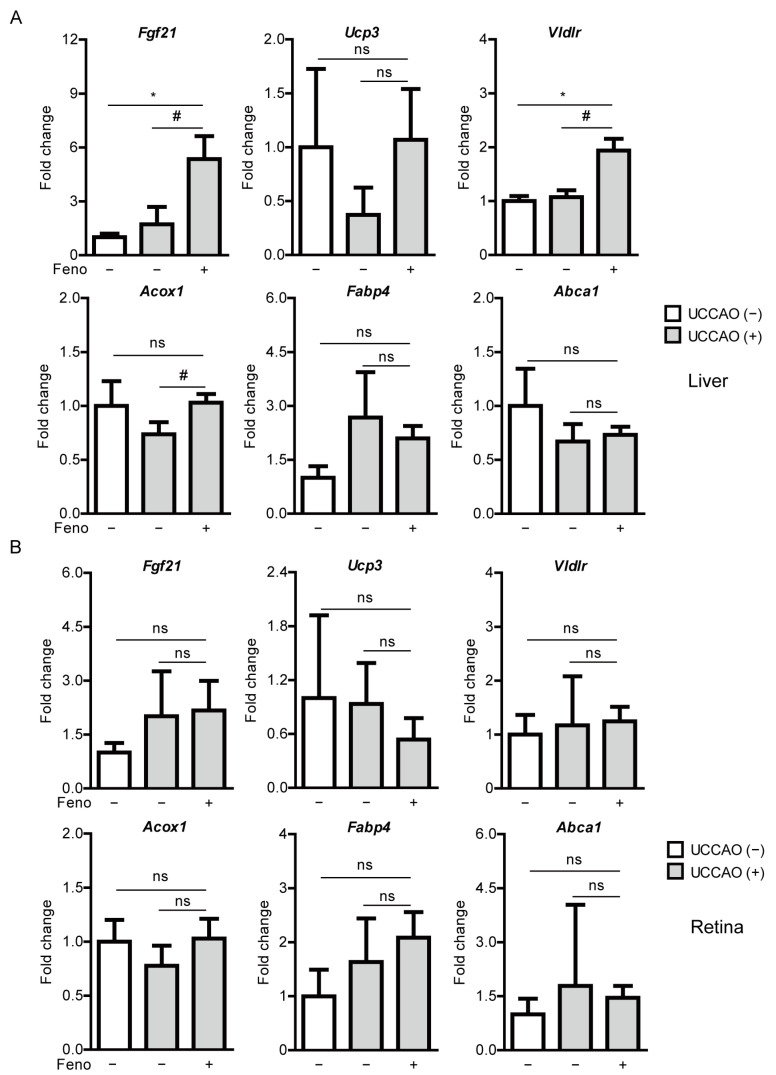
Increases in PPARα downstream gene expressions in the liver by oral administration of fenofibrate in the UCCAO-operated mice. (**A**) Quantitative analyses (*n* = 3 for sham group; 4–5 per UCCAO group) show that oral administration of fenofibrate significantly increased *Fgf21*, *Vldlr,* and *Acox1* expressions in the liver 7 days after UCCAO. (**B**) Quantitative analyses (*n* = 3 for sham group; 4–5 per UCCAO group) show that oral administration of fenofibrate did not change expressions of PPARα downstream gene in the retina. * *p* < 0.05 (one-way ANOVA), # *p* < 0.05 (Student’s *t*-test). The data were analyzed using Student’s *t*-test (two-tailed) or one-way ANOVA followed by a Bonferroni post hoc test and presented as mean ± standard deviation. Feno; fenofibrate. UCCAO; unilateral common carotid artery occlusion.

We examined whether expressions of PPARα downstream genes could be altered by UCCAO. Dramatic changes between the sham-operated liver and the retina and the UCCAO-operated liver and the retina were not observed 7 days after UCCAO (Figure 4). Next, we examined whether expressions of PPARα downstream genes could be upregulated after oral administration of fenofibrate in the UCCAO-operated mice under the same condition. We found that expressions of PPARα target genes (such as *Fgf21*, *Acox1,* and *Vldlr*) were significantly upregulated in the fenofibrate-administered liver, compared with those in the vehicle-administered liver (Figure 4A). The other genes such as *Ucp3*, *Abca1,* and *Fabp4* were still unchanged. There was no significant change in PPARα target gene expressions between the fenofibrate-administered retina and the vehicle-administered retina (Figure 4B).

### 2.5. Fenofibrate Increases Serum FGF21 Levels in a Mouse Model of UCCAO-Induced Ocular Ischemia

**Figure 5:**
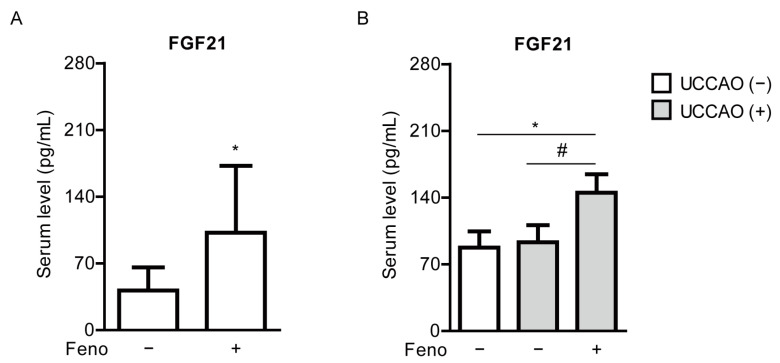
Increases in serum levels of FGF21 by oral administration of fenofibrate in adult mice. (**A**) Quantitative analyses (*n* = 7–8 per group) show that oral administration of fenofibrate significantly increased FGF21 levels in the blood 4 days after oral administration of fenofibrate, right before UCCAO. * *p* < 0.05 (Student’s *t*-test). (**B**) Quantitative analyses (*n* = 6 per group) show that oral administration of fenofibrate increased FGF21 levels in the blood 12 days after UCCAO. * *p* < 0.05 (one-way ANOVA), # *p* < 0.05 (Student’s *t*-test). The data were analyzed using Student’s *t*-test (two-tailed) or one-way ANOVA followed by a Bonferroni post hoc test and presented as mean ± standard deviation. Feno; fenofibrate. UCCAO; unilateral common carotid artery occlusion.

We examined whether fenofibrate could increase serum FGF21 levels. Increases in serum FGF21 levels by fenofibrate treatment or other PPARα agonists have been reported in various types of experimental models [35,36] and clinical studies [37,38,39]. As expected, serum levels of FGF21 were significantly elevated in the fenofibrate-administered mice, in comparison with those of the vehicle-administered mice on 4 days after oral administration of fenofibrate, right before induction of UCCAO (Figure 5A). Furthermore, increased serum FGF21 levels were seen in the fenofibrate-administered UCCAO-operated mice at the endpoint of the experiment, on day 12 after UCCAO, in comparison with the sham-operated mice or the vehicle-administered UCCAO-operated mice (Figure 5B).

### 2.6. Fenofibrate Suppresses Stabilization of Hypoxia-Inducible Factor in a Mouse Model of UCCAO-Induced Ocular Ischemia

**Figure 6:**
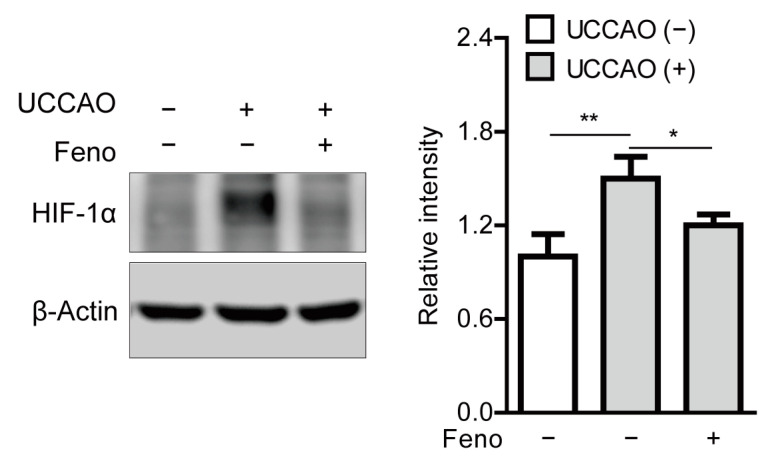
Reduction in stabilization of retinal hypoxia-inducible factor-1α (HIF-1α) by oral administration of fenofibrate in the UCCAO-operated mice. A representative image and quantitative analysis (*n* = 4–5 per group) show that oral administration of fenofibrate significantly suppressed HIF-1α stabilization in the retina 8 h after UCCAO. * *p* < 0.05, ** *p* < 0.01. The data were analyzed using one-way ANOVA followed by a Bonferroni post hoc test and presented as mean ± standard deviation. Feno; fenofibrate. UCCAO; unilateral common carotid artery occlusion.

Previously, it was reported that UCCAO could induce stabilization of hypoxia-inducible factor-1α (HIF-1α) in the retina [14]. Therefore, we examined whether this finding could be affected by oral administration of fenofibrate. Stabilized HIF-1α expression was significantly observed in the retina 8 h after UCCAO, and its expression significantly decreased in the fenofibrate-administered retina, in comparison with that in the vehicle-administered retina (Figure 6).

### 2.7. Fenofibrate Modulates Hypoxia-Responsive Gene Expressions in a Mouse Model of UCCAO-Induced Ocular Ischemia

**Figure 7:**
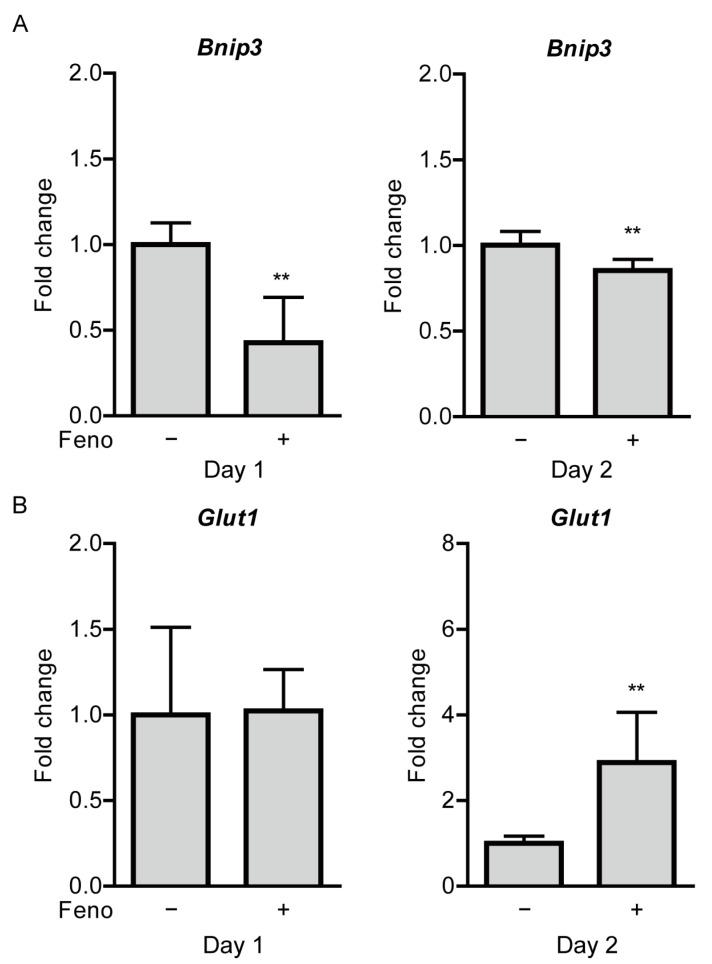
Modulation of retinal hypoxia-responsive gene expressions by oral administration of fenofibrate in the UCCAO-operated mice. (**A**) Quantitative analyses (*n* = 4–7 per group) show that oral administration of fenofibrate significantly reduced increases in expressions of *Bnip3* in the retina 1 and 2 days after UCCAO. (**B**) Quantitative analyses (*n* = 4–7 per group) show that oral administration of fenofibrate increased *Glut1* expressions in the retina 2 days after UCCAO. ** *p* < 0.01. The data were analyzed using Student’s *t*-test (two-tailed) and presented as mean ± standard deviation. Feno; fenofibrate. UCCAO; unilateral common carotid artery occlusion.

**Figure A7:**
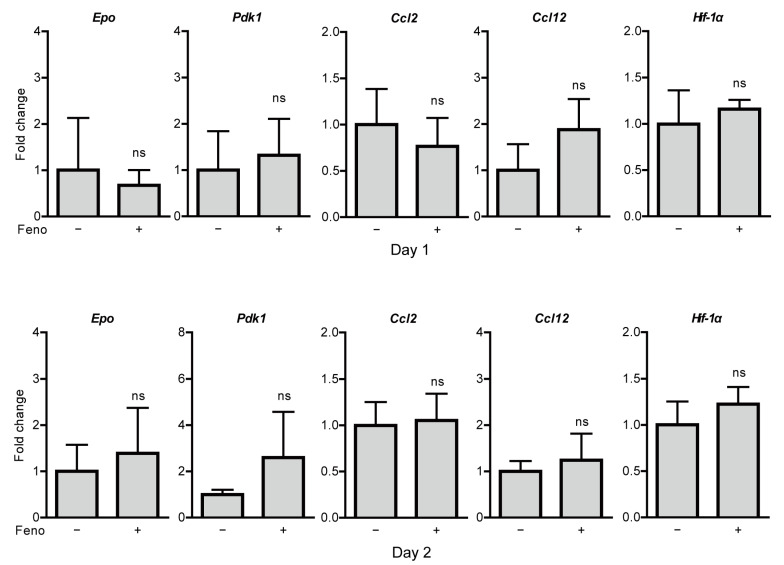
No change in retinal hypoxia-responsive gene expressions by oral administration of fenofibrate in the UCCAO-operated mice. Quantitative analyses (*n* = 4 per group, upper) show that oral administration of fenofibrate did not change expressions of *Epo*, *Pdk1*, *Ccl2, Ccl12,* and *Hif-1α* in the retina 1 day after UCCAO. Quantitative analyses (*n* = 5–7 per group, lower) show that oral administration of fenofibrate did not change expressions of *Epo*, *Pdk1*, *Ccl2*, *Ccl12* and *Hif-1α* in the retina 2 days after UCCAO. *p* > 0.05. The data were analyzed using Student’s *t*-test (two-tailed), and presented as mean ± standard deviation. UCCAO; unilateral common carotid artery occlusion.

We examined whether hypoxia-responsive gene expressions in the ischemic retina are changed after oral administration of fenofibrate. Increases in *Bnip3* expressions were reduced in the fenofibrate-administered UCCAO-operated retina, in comparison with those in the vehicle-administered UCCAO-operated retina 1 and 2 days after UCCAO (Figure 7A). Interestingly, significantly upregulated *Glut1* expression was seen in the fenofibrate-administered UCCAO-operated retina, in comparison with that in the vehicle-administered UCCAO-operated retina 2 days after UCCAO (Figure 7B). The other hypoxia-responsive gene expressions studied above (Figure 1C) were not altered in the fenofibrate-administered UCCAO-operated retina (Figure A7).

## 3. Discussion

In our current study, we demonstrated administration of a PPARα agonist, fenofibrate, protected retinal function in the UCCAO-operated mice via modulating hypoxia-responsive genes. Furthermore, significant increases in PPARα target gene expressions in the liver and elevation of serum FGF21 levels were detected after treatment of fenofibrate. This report is the first investigation of the development of ocular ischemia by UCCAO and its rescue by PPARα activation together, which is the significant aspect of our study.

In the current data, fenofibrate did not affect the retina directly, evidenced by the results that PPARα target gene expressions were not altered in the fenofibrate-administered retina. In contrast, fenofibrate affected the liver and increased FGF21 levels in the serum. This finding was in accordance with our previous papers, showing PPARα agonists (fenofibrate or pemafibrate) directly led to induction of PPARα downstream genes in the liver (not in the retina) and led to elevation of serum levels of FGF21 to exert retinal protection in diabetic retinopathy [35] or prematurity of retinopathy [36]. Recently, another group also reported that fenofibrate treatment prevented the development of early diabetic retinopathy without induction of PPARα in the retina in a murine model of type 2 diabetes (specifically, *db/db* mice) [40]. Besides, using the peroxisome proliferator response element-luciferase reporter system, this group demonstrated strong induction of the reporter in the liver by another PPARα agonist GW590735 (not in the retina), suspecting fenofibrate may act systemically via the liver to modulate circulating cytokines, growth factors, or lipids that indirectly affect the retina [40]. FGF21 is a secreted protein that regulates important metabolic pathways in an autocrine/paracrine manner [41,42,43,44]. FGF21 is produced in various tissues, especially in the liver, and circulates to interact with other sites to modulate metabolism [44]. In this regard, we assume that circulating FGF21 in the blood may act on the ischemic retina as a neuroprotective agent. Previously, FGF21 showed therapeutic effects on several models of retinopathies in mice. The first study showed that long-acting FGF21 suppressed neovascularization in mice by suppressing TNF-α expression [45]. The second study showed that long-acting FGF21 maintained retinal function (analyzed by ERG) in Akita and streptozotocin-induced diabetic mice [46]. Finally, long-acting FGF21 reduced retinal vascular leakage in a murine model and in human cells by maintaining claudin-1 expression [47]. In the central nervous system (especially, the brain), recombinant human FGF21 improved ischemic outcomes in a murine model of stroke via alleviation of neuroinflammation [48]. Delayed recanalization after middle cerebral artery occlusion improved neurological deficits in rats via increasing endogenous FGF21 expression, followed by attenuation of neuronal death in penumbra [49]. Taken together, although more studies are needed to determine whether FGF21 exerts cellular protection in the ischemic retina, fenofibrate-induced circulating FGF21 may have protective effects on the UCCAO-induced ischemic retina.

Based on our data, an increase in *Glut1* expression was shown in the fenofibrate-administered retina. FGF21 exerts a therapeutic effect on glucose and lipid metabolism in mice [50]. Therefore, a phase 2B clinical trial has been investigated to examine whether pegbelfermin (a novel long-acting FGF21) has therapeutic effects on nonalcoholic fatty liver disease (NAFLD) and nonalcoholic steatohepatitis (NASH) [31]. A previous study demonstrated that FGF21 had a synergistic effect with insulin on glucose absorption that is in accordance with enhanced *Glut1* expression [51]. Another study reported that FGF21 regulated glucose metabolism by increasing mRNA expression of *Glut1* [52]. Upregulation of FGF21 markedly increased *Glut1* mRNA expression in 3T3-L1 adipocytes [53]. Furthermore, FGF21 administration has been shown to exert cardiac protection against ischemia/reperfusion-induced cardiac injuries by upregulation of GLUT1 [54]. Suppressed expression of *Glut1* impaired an entry of glucose into photoreceptors and resulted in a shortage of lipid and glucose fuel in the mouse retina [55]. In this regard, increased serum FGF21 levels may lead to induction of *Glut1*, and this change may bring advantages to the retina via modulation of glucose metabolism against acute ischemic states.

BNIP3 is a membrane-associated protein which is mainly localized in mitochondria [56]. BNIP3 expression has been known to be induced by hypoxia and its upregulation is closely associated with hypoxia-induced cell damages in various cell types [57,58]. In our study, reduction in increased *Bnip3* expression was shown in the fenofibrate-administered retina. Although there should be more studies on whether its reduction is directly or indirectly related to FGF21 or fenofibrate itself, a decrease in *Bnip3* expression at least implies that retinal hypoxic insults by UCCAO could be ameliorated by administration of fenofibrate.

PPARα agonists have been reported to reduce activation of HIF-1α in ocular ischemia. Retinal HIF-1α expression increased in a murine model of oxygen-induced retinopathy and its expression decreased by treatment of fenofibric acid, an active form of fenofibrate [27,59]. In PPARα knockout mice, fenofibric acid treatment could not reduce upregulated expression of HIF-1α in the ischemic retina of oxygen-induced retinopathy [27]. This implies that its effect of fenofibric acid is PPARα-dependent. Another study demonstrated that increased immunoreactivities of HIF-1α were reduced by fenofibrate treatment in the ischemic retina of oxygen-induced retinopathy [60]. In our previous study, administration of pemafibrate, a selective PPARα modulator, suppressed an increase in immunoreactivities of HIF-1α in the ischemic retina of oxygen-induced retinopathy [36]. Furthermore, we demonstrated that elevated serum FGF21 levels by pemafibrate administration may inhibit retinal HIF activation through the in vitro HIF-reporter luciferase assay using long-acting FGF21 and pemafibrate itself under a CoCl_2_-induced pseudo-hypoxic condition [36]. Our current study showed that fenofibrate treatment could also reduce HIF-1α induction in the ischemic retina, as one of similar characteristics of PPARα agonists. It has not been clearly defined whether elevated serum FGF21 levels by fenofibrate treatment or fenofibrate itself exerts a reducing effect for HIF-1α activation in the ischemic retina. Although more studies are needed for this issue, retinal hypoxic injuries by UCCAO could be at least ameliorated by treatment of fenofibrate.

Based on our ERG data, fenofibrate rescued reduction in the amplitude of b-wave induced by UCCAO. This implies that fenofibrate treatment may affect the inner retinal layer, as b-wave mainly reflects a physiological condition in the inner retinal layer [61]. Additionally, we found that UCCAO decreased retinal synaptophysin expression and its reduction was slightly suppressed by fenofibrate treatment. Synaptophysin is one of the synaptic vesicle membrane proteins and has an important role in a neuronal network which could be associated with retinal function [62,63]. Reduction in synaptophysin expression was also seen in other experimental retinopathy models (streptozotocin-induced diabetic retinopathy [64] and bilateral common carotid artery occlusion-induced ischemic retinopathy [17]). Although it has not been clearly unraveled that reduction in synaptophysin expression directly contributes to retinal dysfunction, we showed synaptic functional damages in the retina could be induced by UCCAO and fenofibrate administration may partially ameliorate these damages. In fact, other potent synaptic proteins may also be cooperatively involved in suppression of UCCAO-induced retinal dysfunction by oral administration of fenofibrate. Further studies may be needed for this issue.

Previously, inner retinal thinning was described in the UCCAO-operated mice at the chronic stage [14]. However, we could not detect acute and chronic retinal thinning in our preliminary experiment. It has been suggested that inner retinal thinning could occur more than 10 weeks after UCCAO [14]. In our study, we could not follow up changes in retinal thickness until 10 weeks after UCCAO, which could be one of possible reasons for its discrepancy. In fact, we primarily focused on a retinal functional change at the acute stage after UCCAO and its rescue by fenofibrate administration in this study. This is because of a technical difficulty of using oral gavages for chronic repetitive drug treatments. To properly investigate the chronic aspect of ocular ischemia by UCCAO and its rescue by fenofibrate administration, a better method for oral administration (especially supplementing diet with fenofibrate) needs to be applied, of which way is safer for experimental models and more stable in maintaining the effects of fenofibrate for the models. This will be considered for our further work.

There were several limitations to our study. Dissolving fenofibrate was extremely difficult even though we used 1% DMSO with PBS. This low solubility (fenofibrate: a poorly water-soluble drug [65]) could increase variations in the amount of fenofibrate administered to each mouse, and a high concentration of DMSO may be toxic to mice. This matter could also be addressed by the oral administration method of a supplement diet. Next, we administered fenofibrate before UCCAO, and kept supplying fenofibrate after UCCAO. In this condition, pre-and post-effects of fenofibrate could be intermingled. Pre- and post-effects of fenofibrate may need to be divided in further studies. Moreover, although we used fenofibrate administration for 4 consecutive days before UCCAO to boost protective effects of fenofibrate and its continuous administration once every 2 days until the end of the experiment after UCCAO to maintain the effects of fenofibrate, this approach to treatment has not been clearly optimized for protective effects of fenofibrate against UCCAO-induced ocular ischemia. This may be one of possible reasons that we could not detect upregulation of several PPARα target genes in the liver by administration of fenofibrate. It is still challenging to define how many days of administration and which ranges of concentrations of fenofibrate are the most effective for prevention of ocular ischemia. Besides, direct effects of FGF21 on this model have not been investigated yet. Finally, UCCAO could evoke massive effects in the surgical sites by its invasive method for the model development. As the midline of the neck is incised and sutures are permanently placed to occlude the carotid artery, inflammation or its associated unexpected outcomes could occur in the surgical sites or in the body. These issues could be partially addressed in that the surgical steps were conducted for all experimental mice. Nonetheless, therapeutic effects of fenofibrate in the surgical sites or in the body could not be excluded as fenofibrate has systemic effects on metabolism. Taken together, we need further studies to clarify those mechanisms.

Recently, a new neuroprotective drug (KUS121) has been under clinical trial for CRAO [66]. CRAO brings acute damages to nerve cells caused by arterial occlusion, and KUS121 is expected to inhibit dysfunction of intracellular organelles, mainly the smooth endoplasmic reticulum, and maintain their activities. Its treatment in nine CRAO eyes had improvement in visual acuity, and no serious complications were observed. However, this KUS121 study is still in phase 1/2 clinical trial, so this drug may take long time to be utilized for CRAO treatment. In the aspect of drug availability, fenofibrate (already used in clinic) is ready to use at this point. This implies that fenofibrate has a considerable advantage of being easily applicable in clinic for CRAO treatment.

In conclusion, our current work was preliminarily prepared to build groundwork for future research using oral supplement diet with fenofibrate as a therapeutic drug in a mouse model of UCCAO-induced ocular ischemia. Although further studies will be needed for accumulating evidence, we shortly suggest a promising fenofibrate therapy by induction of neuroprotective FGF21 and suppression of retinal dysfunction in carotid artery occlusion-induced ischemic retinopathy as one type of human CRAO.

## 4. Materials and Methods

### 4.1. Animal

Mice (6- to 7-week-old male C57BL/6) were obtained from CLEA Japan (Tokyo, Japan) and freely supplied with food and water under a 12 h light–dark cycle in a temperature-controlled room. All protocols for animal experiments were approved by the Ethics Committee on Animal Research of the Keio University School of Medicine (Approved number #16017/2020). All procedures were followed by the international standards of animal care and use, Animal Research: Reporting in Vivo Experiments (ARRIVE) guidelines (last accessed date: 25 January 2021, http://www.nc3rs.org.uk/arrive-guidelines) and the ARVO Statement for the Use of Animals in Ophthalmic and Vision Research.

### 4.2. A Murine Model of UCCAO-Induced Ocular Ischemia and Oral Administration of Fenofibrate

After random grouping, mice were orally administered vehicle (1% DMSO dissolved PBS) or fenofibrate (60 mg/kg, 1% DMSO dissolved PBS) once a day for 4 days before UCCAO. A mouse model of UCCAO-induced ocular ischemia was developed, as previously described [14]. Briefly, under deep anesthesia with a combination of midazolam (40 μg/100 μL; Sandoz, Tokyo, Japan), medetomidine (7.5 μg/100 μL; Orion, Espoo, Finland), and butorphanol tartrate (50 μg/100 μL; Meiji Seika Pharma, Tokyo, Japan) [67], the midline of the mouse neck was incised to find the common carotid artery, and then the common carotid artery in the right side was ligated by 6−0 silk sutures. Incised wounds of the mouse were sutured and then the mouse was recovered. After UCCAO, administration of fenofibrate was performed once every 2 days until the end of each experiment. Body weight was measured during the experimental period.

### 4.3. Optical Coherence Tomography (OCT)

OCT (Envisu R4310, Leica, Wetzlar, German) was conducted as previously described [19]. Briefly, mice were subjected to mydriasis by a combination of 0.5% tropicamide and 0.5% phenylephrine (Santen Pharmaceutical, Osaka, Japan). After 5 min, mice were anesthetized with a combination of midazolam (40 μg/100 μL; Sandoz, Tokyo, Japan), medetomidine (7.5 μg/100 μL; Orion, Espoo, Finland), and butorphanol tartrate (50 μg/100 μL; Meiji Seika Pharma, Tokyo, Japan). Anesthetized mice were placed in an OCT platform and subjected to OCT analyses. B-scan images were obtained from equatorial slices of en-face scans and the retina was examined at 0.2, 0.4, and 0.6 mm from the optic nerve head. Retinal thickness was measured from the outer retina to the inner retina including the ganglion cell layer. Total retinal thickness: the outer retina to the inner retina including the ganglion cell layer; Inner retinal thickness: the inner retina including the ganglion cell layer; Outer retinal thickness: the outer retina.

### 4.4. Electroretinography (ERG)

ERG was conducted as previously described [19,68]. Briefly, mice were placed in a dark room for more than 12 h for dark adaptation. Then, mice were anesthetized with a combination of midazolam (40 μg/100 μL; Sandoz, Tokyo, Japan), medetomidine (7.5 μg/100 μL; Orion, Espoo, Finland), and butorphanol tartrate (50 μg/100 μL; Meiji Seika Pharma, Tokyo, Japan). A heating pad was placed under the mice throughout the experiment. Pupils were dilated with one or two drops of a mixture of 0.5% tropicamide and 0.5% phenylephrine (Santen Pharmaceutical, Osaka, Japan). Recording of scotopic ERG responses was processed by using a Ganzfeld dome and LED stimulators with an acquisition system (PuREC, MAYO, Inazawa, Japan). The active electrodes were softly touched on the contact lens of the mice and a reference electrode was inserted into the mouth. A clipping electrode was placed around the tail on the ground. The amplitudes of a-wave (from the baseline to the lowest point of a-wave) and b-wave (from the lowest point of a-wave to the peak of b-wave) were measured with various light stimuli.

### 4.5. Measurement of Serum FGF21 Levels

After blood sample collection as previously described [35,36], serum samples were added to a 96-well plate provided from an FGF21 ELISA kit (Cat #RD291108200R, BioVendor Laboratory Medicine, Brno, Czech Republic). Serum levels of FGF21 in the samples were measured, following the manufacturer’s instructions.

### 4.6. Quantitative PCR

Quantitative PCR was conducted, as previously described [35,36,68]. Briefly, the RNA of the retina and the liver was extracted by using a RNeasy Plus Mini Kit (Qiagen, Velno, Netherlands). RT-PCR was conducted using a ReverTra Ace^®^ qPCR RT Master Mix with gDNA Remover (TOYOBO, Osaka, Japan), and Real-time PCR was conducted using a THUNDERBIRD^®^ SYBR^®^ qPCR Mix (TOYOBO, Osaka, Japan) with the Step One Plus Real-Time PCR system (Applied Biosystems, Waltham, MA, USA). Primers that we used are listed in Table 1. The fold alteration between levels of different transcripts was calculated by the ΔΔCT method.

### 4.7. Western Blotting

Extraction of total retinal protein and electrophoresis followed by visualization of target bands were conducted as same as described in our previous papers [19,35,36,68,69]. Primary antibodies that we used in this study are anti-HIF-1α (1:500, Cat #36169, Cell Signaling Technology, Danvers, MA, USA), anti-synaptophysin (1:1000, Cat #SAB4502906, Sigma, Tokyo, Japan), and anti-β-Actin (1:5000, #3700, Cell Signaling Technology, USA). For visualization of the bands, HRP-conjugated secondary antibodies (1:1000 for anti-HIF-1α and anti-synaptophysin; 1:5000 for anti-β-Actin, GE Healthcare, Chicago, IL, USA) were used. Intensities of the bands were quantified via NIH ImageJ software (National Institutes of Health, Bethesda, MD, USA).

### 4.8. Statistical Analysis

Data were analyzed with GraphPad Prism 5 (GraphPad Software, San Diego, CA, USA). Statistically significant difference was calculated by using Student’s *t*-test, one-way ANOVA followed by a Bonferroni post hoc test, or two-way ANOVA followed by a Bonferroni post hoc test depending on the data set. Any p-values of less than 0.05 were considered statistically significant.

## Figures and Tables

**Table 1:** Primer list.

Name	Direction	Sequence (5′→3′)	Accession Number
*Hprt*	Forward	TCAGTCAACGGGGGACATAAA	NM_013556.2
	Reverse	GGGGCTGTACTGCTTAACCAG	
*Epo*	Forward	GGCCATAGAAGTTTGGCAAG	NM_007942
	Reverse	CCTCTCCCGTGTACAGCTTC	
*Bnip3*	Forward	GCTCCCAGACACCACAAGAT	NM_009760.4
	Reverse	TGAGAGTAGCTGTGCGCTTC	
*Pdk1*	Forward	GGCGGCTTTGTGATTTGTAT	NM_172665.5
	Reverse	ACCTGAATCGGGGGATAAAC	
*Glut1*	Forward	CAGTTCGGCTATAACACTGGTG	NM_011400.3
	Reverse	GCCCCCGACAGAGAAGATG	
*Ccl2*	Forward	CCCAATGAGTAGGCTGGAGA	NM_011333.3
	Reverse	TCTGGACCCATTCCTTCTTG	
*Ccl12*	Forward	GCTACAGGAGAATCACAAGCAGC	NM_011331.3
	Reverse	ACGTCTTATCCAAGTGGTTTATGG	
*Ucp3*	Forward	GGAGTCTCACCTGTTTACTGACAACT	NM_009464.3
	Reverse	GCACAGAAGCCAGCTCCAA	
*Abca1*	Forward	CGTTTCCGGGAAGTGTCCTA	NM_013454.3
	Reverse	GCTAGAGATGACAAGGAGGATGGA	
*Fabp4*	Forward	CCGCAGACGACAGGA	NM_024406.3
	Reverse	CTCATGCCCTTTCATAAACT	
*Fgf21*	Forward	AACAGCCATTCACTTTGCCTGAGC	NM_020013.4
	Reverse	GGCAGCTGGAATTGTGTTCTGACT	
*Vldlr*	Forward	GAGCCCCTGAAGGAATGCC	NM_001161420.1
	Reverse	CCTATAACTAGGTCTTTGCAGATATGG	
*Acox1*	Forward	TCTTCTTGAGACAGGGCCCAG	AF006688.1
	Reverse	GTTCCGACTAGCCAGGCATG	
*Hif-1α*	Forward	GGTTCCAGCAGACCCAGTTA	NM_001313919.1
	Reverse	AGGCTCCTTGGATGAGCTTT	

## Data Availability

The data presented in this study are available on request from the corresponding author.
